# Increased incidence of rare codon clusters at 5' and 3' gene termini:implications for function

**DOI:** 10.1186/1471-2164-11-118

**Published:** 2010-02-18

**Authors:** Thomas F Clarke, Patricia L Clark

**Affiliations:** 1 Department of Chemistry and Biochemistry, University of Notre Dame, Notre Dame, IN 46556, USA

## Abstract

**Background:**

The process of translation can be affected by the use of rare versus common codons within the mRNA transcript.

**Results:**

Here, we show that rare codons are enriched at the 5' and 3' termini of genes from *E. coli *and other prokaryotes. Genes predicted to be secreted show significant enrichment in 5' rare codon clusters, but not 3' rare codon clusters. Surprisingly, no correlation between 5' mRNA structure and rare codon usage was observed.

**Conclusions:**

Potential functional roles for the enrichment of rare codons at terminal positions are explored.

## Background

The amino acid sequence of a protein is determined by the sequence of trinucleotide codons in its mRNA. The 20 most common amino acids are encoded by 61 different codons. With the exception of methionine and tryptophan, all of these amino acids are encoded by multiple codons, meaning that many different nucleotide sequences can encode an identical protein sequence. However, the selection of a particular coding sequence is not random. Instead, as a result of numerous forces, including GC bias, some codons are used more frequently than others. The higher demand for these common codons correlates with an increased production of their cognate tRNAs, leading to faster [1-3] and more accurate [4,5] translation of common codons relative to their rare counterparts.

Yet if rare codons persist only due to incomplete selection against the associated lower translational fidelity and protein yield, it would be expected that rare codons would be randomly distributed throughout the open reading frames (ORFs) of the genome. However, this is not the case. Instead, rare codons often appear in large clusters [6]. These clusters can cause translational pausing, which reduces the local protein translation rate. Rare codon clusters have been identified in genes of all functional classes in a wide variety of organisms [6].

The clustering of rare codons indicates that there are forces that influence the selection of rare codons within mRNA sequences. It has been suggested that rare codons could influence co-translational protein folding. For example, pausing synthesis of the nascent polypeptide chain could allow folding events to occur at protein domain boundaries, or for slower folding secondary structures [7-10]. However, other factors could also contribute to positive selection for rare codons within an mRNA sequence. For example, stable mRNA secondary structure, especially within the first 40 nucleotides at the 5' end of an open reading frame, could negatively affect protein expression by limiting access to the ribosome binding site or initiator methionine codon [11]. For some sequences, strategic placement of one or more rare codons could disrupt 5' mRNA secondary structure. In this case, selective pressures against rare codons would be balanced by the selective pressure against mRNA structure, causing an enrichment of rare codons beyond what would be expected by random chance. It has also been suggested that, for genes encoding proteins bearing N-terminal signal sequences, 5' rare codons could have a functional role related to secretion, perhaps by transiently reducing translation rate prior to membrane localization of the nascent chain [12]. Though there have been fewer discussions of possible beneficial roles for 3' rare codon clusters, these clusters could cause nascent polypeptide chains to dwell at the ribosome surface near the end of translation [13], which could allow for the association of molecular chaperones, other subunits of a multimeric protein, partner proteins, or factors involved in targeting or degradation [14].

Here, we examine the abundance of rare codon clusters at gene termini and other locations, revealing an enrichment of rare codons at both the 5' and 3' end of ORFs from *E. coli *and other prokaryotes. We examine possible roles for these rare codon clusters in protein biogenesis.

## Results

To quantify the relative rareness of codons used across an entire ORFeome, we used the previously developed %MinMax algorithm [6,15]. %MinMax determines the relative commonness or rareness of an mRNA sequence, given the constraints of the underlying protein sequence and the relative abundances of the codons in a particular organism. In contrast to %MinMax, other methods to quantify codon usage have focused on the relative commonness of codons [16], which is useful for estimating expression levels but is not designed for investigating the presence of rare codons or translation rate. Similarly, methods that use intracellular tRNA concentrations to estimate translation speed [17,18] are limited to the small number of organisms with measured tRNA concentrations and must take additional measures to account for the differences in translation speed for tRNAs that bind to multiple codons, such as the 3.4-fold difference in translation rates by the same tRNA for the glutamic acid codons GAA versus GAG [19].

**Figure 1:**
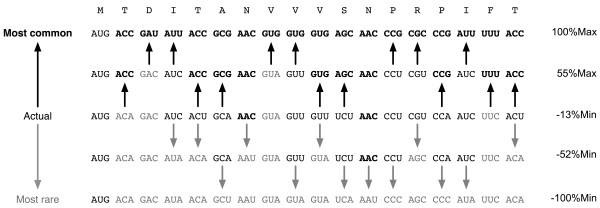
**Effects of synonymous codon substitutions on %MinMax**. The %MinMax calculation was applied to a series of synonymous sequences encoding the N-terminal 18 amino acids of *Salmonella *phage P22 tailspike. The first 18 codons of the wild type tailspike mRNA sequence (middle line) can be changed to common codons (bold text) or rare codons (gray text), resulting in dramatic differences in the %MinMax output, without altering the underlying amino acid sequence.

**Figure 2:**
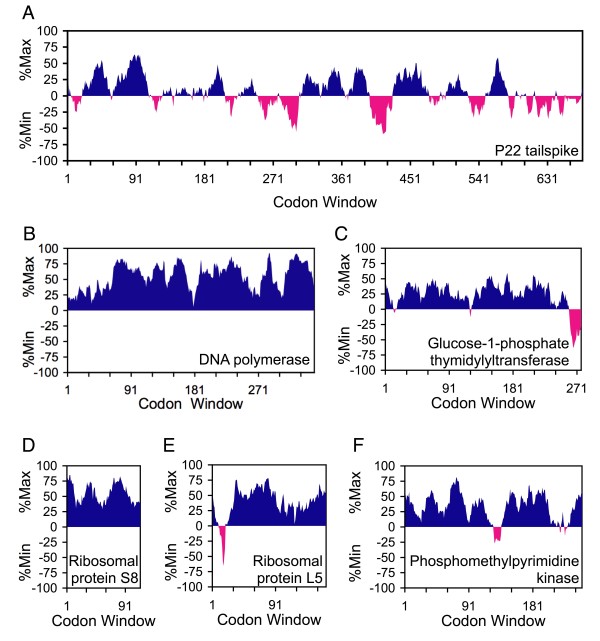
**%MinMax outputs for a variety of genes**. The *Salmonella *phage P22 tailspike gene (A) is highly expressed but contains significant rare codon clusters. Genes in the *E. coli *ORFeome can contain no rare codon clusters (B, D), 5' rare codon clusters (E), 3' rare codon clusters (C) or central rare codon clusters (F).

To evaluate the relative rareness of any given mRNA sequence, the %MinMax algorithm compares the codon usage frequency of the sequence to the usage frequency for theoretical sequences encoding the same amino acid sequence using the most common or most rare codons (see Methods). The average usage frequency of all codons encoding each amino acid in the sequence is used as a baseline (0%Min/Max). Sequences that are encoded using codons that are less common than average produce a %Min value of up to -100%Min, while sequences that are encoded using more common codons produce a %Max value of up to 100%Max. Figure 1 provides an example of how synonymous codon substitutions from rare to common (or vice versa) will change the %MinMax output. The %MinMax output for a selection of *E. coli *genes is shown in Figure 2. Genes are evaluated using a sliding window, typically 18 codons long, to identify clusters of codons that are common or rare (Figure 2). Incomplete windows are not considered, so that a gene of length *n *begins at window 1 (covering codons 1-18) and ends at window *n*-18 (covering codons *n*-18 to *n*). %MinMax results for the actual mRNA sequences are compared to random reverse translations, i.e. synonymous gene sequences created through random selection of codons from a weighted codon usage database [6].

The %MinMax algorithm identified significant enrichment of %Min windows relative to a random distribution of codons, indicating significant clustering of rare codons throughout the ORFeomes of several organisms, including *E. coli, H. sapiens, A. thaliana *and *S. cerevisiae *[6]. %MinMax can be performed on any sequence from any organism with enough sequence data to accurately determine codon usage frequencies. The rare codon clusters identified with %MinMax correlate with experimentally determined translation pause sites [6].

**Figure 3:**
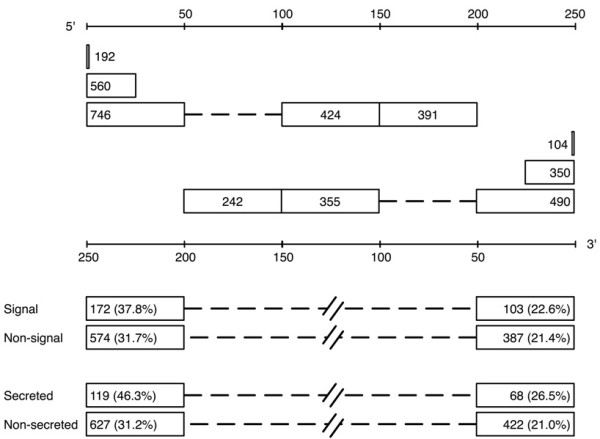
**The number of *E. coli *genes containing a rare codon cluster for a given sequence range or condition**. The number of genes of the 2622 greater than 250 windows with a rare codon cluster in the terminal window, within 25 windows of the terminus and within 50 windows of the terminus are shown from the 5' end (top axis) or 3' end (bottom axis). As controls, two non-terminal ranges are also shown for the 50 window frame. The distribution for genes with versus without signal sequences and predicted versus not predicted to be secreted are also shown.

Here, we used %MinMax to examine the locations of rare codon clusters within the primary structure of genes across the *E. coli *ORFeome. In *E. coli*, codon windows that score -10%Min or more rare represent a significant enrichment of rare codons [6], so -10%Min was used as a cut-off to identify rare codon clusters. To identify those clusters specifically associated with either the 5' or 3' terminus of a gene, versus non-terminal effects, we examined only those genes with at least 250 %MinMax windows (≥ 268 codons). For *E. coli*, this represents 2,262 of the total 4,288 genes in the ORFeome [20]. Of these 2,262 genes, 1,511 (66.8%) include at least one rare codon cluster of at least -10%Min (Figure 3). At the 5' end, 746 (33.0% of total) genes have a rare codon cluster in the first 50 windows, meaning that nearly half of the genes longer than 250 windows with a rare codon cluster have a rare codon cluster in the first 50 windows. Furthermore, 560 genes have a rare codon cluster in the first 25 windows (corresponding to 24.7% of the total dataset, and 37% of genes longer than 250 windows with a rare codon cluster). By comparison, there are fewer rare codon clusters at non-terminal positions: only 424 genes (16.2% of the total data set) have a rare codon cluster between windows 101 and 150 and only 391 (17.3%) have a rare codon cluster between windows 151 and 200.

We also examined the prevalence of rare codon clusters at 3' gene termini. At the 3' end, 490 genes (21.6% of total) have a rare codon cluster within the last 50 windows and 350 genes (15.5% of total) have a rare codon cluster within the last 25 windows (Figure 3). By comparison, there are 355 (15.7%) and 242 (10.7%) genes with rare codon clusters between windows 101-150 and 151-200 from the 3' terminus respectively (p < 0.0001 in both cases, Fischer's exact, two-tailed test).

**Figure 4:**
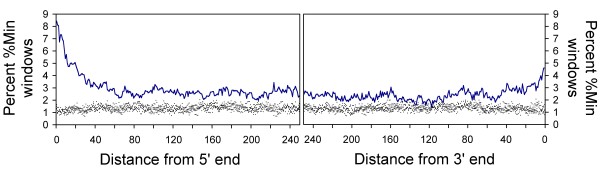
**Enrichment of rare codon clusters at the 5' and 3' termini of *E. coli *open reading frames**. At non-terminal positions, 2.35% of ORFs contain a rare codon cluster. In contrast, 8.49% of ORFs contain a rare codon cluster at the 5' end, and 4.60% of ORFs contain a cluster at the 3' terminus. Only ORFs ≥ 250 windows long were evaluated. Random reverse translations (black dots) of the *E. coli *ORFeome show no position-specific variation in the percent of ORFs containing a rare codon cluster.

The enrichment of rare codons in the 25 or 50 windows near gene termini could result from an enrichment of rare codons just at the terminal window, indicating a selection limited to the area immediately adjacent to the start or stop codon, or from a broader region of selection for rare codon clusters. To address this, we examined the percentages of *E. coli *genes longer than 250 windows that have a rare codon cluster at each individual window position (Figure 4). At non-terminal positions, defined here as ≥ 50 windows from the 5' or 3' terminus, an average of only 2.35% individual 18-codon windows are -10%Min or more rare. At the termini, the picture is quite different: 8.49% of genes begin with a rare codon cluster and 4.60% conclude with a rare codon cluster. At the 5' end, the higher population of rare codon clusters at discrete positions is not limited to only the first few windows; positions up to 22 windows from the start codon show significant (greater than 7σ) enrichment of rare codon clusters relative to random reverse translations.

We also investigated whether the rare codon clustering observed at either terminus was due to any particular rare codon or subset of rare codons (Additional File 1, Figure S1). Briefly, for 5' rare codon clusters, genes were separated into two groups: those with 5' rare codon clusters and those without 5' rare codon clusters. The codon usage for the first 50 codons, the final 50 codons, and the interior codons was tallied. Two separate 2 × 2 contingency tables were then constructed, using the terminal codon usage (5' in one table, 3' in the other) and interior codon usage as columns and the genes with 5' rare codon clusters and without 5' rare codon clusters as rows. A chi-square with Yates correction was used to calculate the p-value for the distribution. This process was repeated for genes with 3' rare codon clusters, predicted signal sequences, and predicted secreted genes. The 5' codon usage for genes predicted to be secreted or predicted to have a signal sequence shows statistically significant enrichment for certain codons; however, this primarily reflects amino acid usage, not codon selection. Tryptophan, aspartic acid, asparagine, glutamine and tyrosine are all under-represented in signal sequences, while cysteines and alanines are over-represented. There is under-enrichment of the most common glycine, arginine and threonine codons, though no rare codons are specifically over-enriched.

There were also some specific and statistically significant correlations that came from the analysis of 5' rare codon clusters: the two common glycine codons, the second most common arginine codon, the most common glutamic acid codons, the most common threonine codon, the most common valine codon and the most common leucine codon were all under-enriched at the 5' terminus in genes with 5' rare codon clusters. This should not be surprising, as selecting for a subset of genes with rare codons would tend to decrease the number of very common codons. No specific subset of rare codons was enriched, however, indicating that the effect is not for a specific subset of codons but rather for the quality of rareness independent of any particular amino acid or codon. At the 3' terminus, there was enrichment for the rare codons CAA and GCT, though the slight enrichment of only two codons is unlikely to create the broad effect observed here.

While significant enrichment of rare codons extends 22 windows (40 codons) from the 5' end of genes in *E. coli*, the ribosome exit tunnel restricts the conformations and interactions of the 20-40 amino acid residues most recently synthesized by the ribosome [21,22]. Hence, a rare codon cluster near the 5' end of an mRNA sequence would induce translational pausing at positions where little if any of the nascent chain has exited the ribosomal exit tunnel, meaning that a pause at this point would have very little effect on the co-translational folding of the nascent chain [23]. The clustering of rare codons at the 5' end of coding mRNA sequences could instead reflect a selection against mRNA secondary structure, rather than selection related to translation rate and the appearance of the nascent chain. While the ribosome has an intrinsic helicase activity [24] that can unwind mRNA secondary structure in the coding region, 5' secondary structure could obscure the ribosome binding site or interfere with translation initiation.

**Figure 5:**
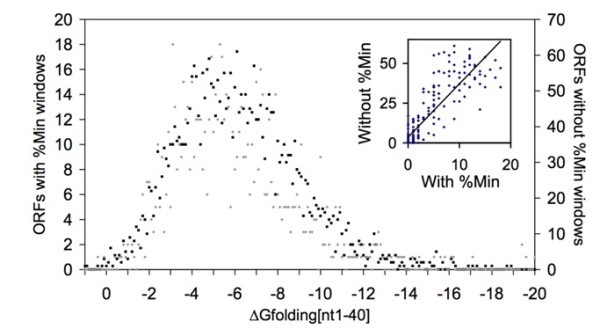
**The population of ORFs with 5' rare codon clusters does not show a dependence on the stability of mRNA folding near the 5' terminus**. The energetics of mRNA folding for nucleotides 1-40 were calculated for every ORF in *E. coli *and the population was separated based on the presence (gray circles, left Y-axis) or absence (black squares, right Y-axis) of a rare codon cluster in the first 13 windows, the windows that would overlap with nucleotides 1-40. The ORFs with or without 5' rare codon clusters have a similar median (ΔG_folding_: -5.6 kcal/mol and -5.9 kcal/mol, respectively) and the same population distribution as seen by the linear regression when comparing the population with rare codons and those without for each ΔG_folding _value (inset).

To address the possibility that a selection against mRNA secondary structure at the 5' end could affect synonymous codon usage, the 5' mRNA stabilities for all *E. coli *genes with rare codon clusters within the first 40 nucleotides (corresponding to the first 13 codon windows) was compared with the mRNA stabilities of all genes that do not have a 5' rare codon clusters (Figure 5). We examined mRNA structure stability in the first 40 nucleotides because others have shown that mRNA stability in this region can correlate with protein expression level [11]. If there is decreased selective pressure against rare codons at the 5' gene termini in order to avoid mRNA secondary structure, genes with rare codon clusters might have corresponding less stable 5' structures. However, there is no significant effect of the presence or absence of rare codons on the thermodynamic stability of the first 40 nucleotides. Genes with or without a 5' rare codon cluster have a similar median and distribution of ΔG_folding _for the first 40 nucleotides (Figure 5, medians of -5.6 kcal/mol and -5.9 kcal/mol, respectively). Regression analysis indicates that the two datasets are linearly related (R^2 ^= 0.7945 with an offset of 0.4 kcal/mol, accounting for the slight difference in medians), indicating that both genes with and without rare codons have the same distribution in mRNA stability. To examine whether the observed offset was significant, the mRNA stabilities for wild type sequences were compared with random reverse translations. If selective pressure against 5' mRNA structure exists, then wild type sequences would be less stable than random reverse translations. For random reverse translations, the distribution of rare versus common codons is random, and hence would not be able to reflect a selection for or against mRNA secondary structure. An offset in stabilities between the random reverse translations with or without rare codon clusters would reflect only the effects of underlying nucleotide bias and not any particular selection. Indeed, the randomly reverse translated genes with rare codons and without rare codons produced the same offset observed for wild type mRNA sequences (medians of -6.1 kcal/mol with rare codon clusters, and -6.6 kcal/mol without), though the randomly reverse translated sequences are slightly more stable than the wild type sequences. The same stability difference was also observed when the ORFeome was randomized using a different strategy [25], which preserves dinucleotide frequencies (medians of -7.0 kcal/mol with rare codon clusters, and -7.6 kcal/mol without). These results indicate that differences in stability between wild type sequences with or without rare codon clusters is a product of nucleotide bias, and not a selection for rare codons.

Approximately 18% of *E. coli *ORFs encode a protein with a predicted N-terminal signal sequence, which is used to transport the encoded protein out of the cell cytoplasm. To examine the influence of signal sequences on the enrichment of rare codon clusters at 5' gene termini, we examined the 454 ORFs longer than 250 windows that are predicted to contain an N-terminal signal sequence (as determined by SignalP 3.0 [26]). Of these 454 genes, 172 (37.8%) contain a rare codon cluster within the first 50 codon windows, compared to 574 (31.7%) of the 1808 genes without a signal sequence. This small but significant enrichment of rare codon clusters (p = 0.0140, Fischer's exact, two-tailed test) is not seen at 3' termini (113 of 478 genes with a signal sequence have a rare codon cluster in the last 50 codon windows, compared to 424 of 1939 without a signal sequence; p = 0.4246). Therefore, the modest enrichment of rare codons is specific for the 5' end of genes with signal sequences, rather than a general position-independent enrichment of rare codon clusters in all genes with N-terminal signal sequences. Genes predicted to be secreted by SecreteomeP [27], an algorithm that searches for motifs originally identified in secreted genes lacking a signal sequence, show a similar enrichment. For the 526 genes classified as secreted with SecreteomeP, there is a significant 5' enrichment of rare codon clusters at 5' gene termini (p < 0.0001), with minimal 3' enrichment of rare codon clusters at 3' gene termini (p = 0.0373) (Figure 3). The SecreteomeP dataset does, however, overlap with the SignalP dataset, with 378 genes appearing in both.

Genes were also examined using their assigned functional categories from the JCVI CMR. Most gene class assignments showed no association with rare codon clusters, either enrichment or under-representation. Hypothetical genes showed a non-specific enrichment of rare codon clusters, with enrichment at all positions (p < 1 × 10^-5^) being reflected in the 5' (p < 1 × 10^-7^) and 3' (p < 1 × 10^-6^) enrichment. Genes assigned to nucleotide (p = 0.0093) and amino acid biosynthesis (p = 0.00025) categories showed a general under-representation of rare codon clusters, as might be expected for categories containing primarily highly expressed genes. The only category that showed a significant orientation-specific effect was energy metabolism, which contained significantly fewer than expected rare codon clusters at the 5' end (p < 1 × 10^-5^), but not in general or at the 3' terminus. The relationship between rare codon clusters and the gene expression level was also examined, using expression levels reported in the NCBI GEO database [28]. Neither the 530 most highly expressed nor the 527 least expressed genes showed any statistically significant correlation to the presence or absence of rare codon clusters in general or at either termini.

In addition to *E. coli*, enrichment of rare codon clusters at the 5' and 3' ends of genes is observed in other prokaryotic ORFeomes. Of 26 prokaryotic ORFeomes examined, 15 showed significant enrichment of rare codon clusters at the 5' terminus and 12 showed significant 3' enrichment (Table 1). While these ORFeomes have varying levels of rare codon clusters over their entire length, the termini are still further enriched beyond the baseline level of rare codon clusters across the entire ORFeome, indicating that the significant enrichment of rare codon clusters is not simply confined to *E. coli *and may reflect general functional roles for rare codon clusters in prokaryotic protein biogenesis. None of the ORFeomes showed a decrease in rare codon clusters at either terminus, versus non-terminal positions.

**Table 1:** The population of rare codon clusters is markedly higher at the termini relative to the non-terminal positions

	Percent of genes with rare codon clusters^*a*^		
	5'	Non-terminal average	3'
*E. coli*	8.49 *	2.35 ± 0.32	4.60 *
*A. tumefaciens*	9.44 *	0.96 ± 0.24	2.87 *
*B. anthracis*	1.08	0.47 ± 0.15	1.03
*B. cereus*	1.21	0.60 ± 0.16	1.33
*B. fragilis*	5.68	3.52 ± 0.37	4.10
*B. subtilis*	4.83	4.04 ± 0.37	5.08
*B. pertussis*	2.01 *	0.14 ± 0.06	0.65 *
*B. melitensis 16 M*	9.97 *	0.86 ± 0.21	3.49 *
*Burkholderia sp. 383*	1.97 *	0.36 ± 0.09	0.72
*C. burnetii*	0.55	0.26 ± 0.17	0.55
*C. neoformans*	11.12	11.06 ± 1.10	21.24 *
*D. radiodurans*	4.54 *	0.92 ± 0.20	2.27
*E. carotovora*	10.28 *	3.59 ± 0.42	6.62 *
*H. pylori*	1.64	0.73 ± 0.31	0.88
*N. meningitidis*	4.69	2.45 ± 0.63	4.59
*Nostoc sp. PCC 7120*	4.22	2.39 ± 0.30	4.26
*P. fluorescens*	3.86 *	0.85 ± 0.16	2.11 *
*R. metallidurans CH34*	2.77 *	0.57 ± 0.14	2.13 *
*S. entericia*	8.49 *	2.47 ± 0.29	4.90
*S. typhimurium*	8.70 *	2.55 ± 0.32	5.19 *
*S. flexneri*	8.28 *	2.63 ± 0.65	6.12
*S. meliloti*	2.72 *	0.42 ± 0.11	1.76 *
*S. aureus*	0.64	0.16 ± 0.11	0.56
*T. thermophilus*	0.73	0.25 ± 0.14	2.02 *
*X. fastidiosa*	7.76 *	3.21 ± 0.63	5.85
*Y. pestis*	9.66 *	3.18 ± 0.83	7.54

^*a *^5' and 3' values that are greater than 7 standard deviations from the average of the non-terminal positions are denoted with an asterisk (*).

The mechanism of translation is very different in eukaryotes versus prokaryotes [29]. Therefore, perhaps not surprisingly, the rare codon enrichment reported above for many prokaryotic organisms is not observed in eukaryotes. The human ORFeome, for instance, shows a decrease in rare codon clusters at 5' gene termini, with the percentage of windows with rare codon clusters dropping from 11.54% at non-terminal positions to 8.14% at the extreme 5' terminus. *Trypanosoma brucei *shows a decrease in rare codon clusters at 3' gene termini (8.6% relative to 9.9% at non-terminal positions). Some genomes, such as *A. thaliana*, show no significant changes at either terminus. *Cryptococcus neoformans *shows a significant 3' increase (21.24% relative to 11.06% at non-terminal positions), though no significant difference is observed at the 5' end.

## Discussion and Conclusions

Determining the role(s) of rare codons in protein biogenesis is complicated by literature reports that describe the negative effects of rare codon clusters, particularly at 5' termini [30], while also reporting examples of rare codons improving protein expression [31], increasing or altering protein activity [32] and being conserved through evolution [33]. Here, we have examined the distribution of rare codons along gene sequences, for different protein classes, in order to identify general forces that could shape rare codon usage.

In the absence of any selection, rare codons and codon clusters would appear randomly throughout the ORFeome. By contrast, our results show that rare codon clusters are more likely to appear at the 5' and 3' ends of *E. coli *genes, rather than non-terminal positions. In particular, genes containing signal sequences showed enrichment at the 5' end of genes, but not the 3' end. This orientation-specific effect suggests a functional usage of rare codons. For example, in eukaryotes, signal recognition particle (SRP) can pause translation of secreted proteins to facilitate their translocation into the endoplasmic reticulum. If SRP is absent, nascent chains are unable to properly engage the translocon, and unprocessed polypeptides accumulate in the cytoplasm [34]. Slowing the rate of translation with antibiotics can counteract the effects of deleting SRP [34]. In a similar manner, it is possible that the prokaryotic 5' rare codon clusters could serve as an alternative route to the same goal: rare codons might represent an SRP-independent mechanism to reduce local translation rates, allowing the ribosome:nascent chain complex to localize to the membrane and facilitating the recognition of exposed signal sequences by the secretion machinery. This process, working in concert with SRP, could increase the efficiency of co-translational or immediate post-translational secretion of nascent polypeptides, preventing the accumulation of transmembrane or secreted polypeptides in the cytoplasm, where their folding and/or secretion may not be as efficient.

Rare codon clusters that occur before any nascent chain sequence has emerged from the ribosomal tunnel could aid secretion via a mechanism independent of signal sequence recognition. For example, if ribosomes bind to an mRNA in rapid succession and are able to translate the sequence without pausing, the resulting polysome will contain multiple ribosomes closely spaced together in both sequence and physical distances [35]. This would increase the local competition for secretory complexes, which could lead to a decrease in secretion efficiency as the accumulating polypeptides are degraded or aggregate during the extended wait for secretion initiation. However, the introduction of a rare codon cluster could space these ribosomes further apart from each other along the mRNA sequence. The ribosomes would be forced to stack up as the first of the group reached the slowly translated section, but after the first ribosome passed through the pause, the second one would enter, slowly translating as the first ribosome more rapidly translated the more common downstream sequence, and this process would repeat for all subsequent ribosomes. This staggering of ribosomes with rare codon clusters could potentially alleviate local competition for translocation complexes and increase the efficiency of secretion.

We also examined an alternative explanation for positive selection of 5' rare codon clusters. Stable mRNA secondary structure can inhibit protein expression by interfering with the initiation of translation, and it has been suggested that rare codons might be employed at the 5' terminus to destabilize these structures [11]. Yet a comparison of ORFs containing rare codon clusters at the 5' end versus those without clusters revealed that potential mRNA secondary structure is independent of rare codon clusters; the distribution of thermodynamic stabilities is similar in both sets of genes. Indeed, while it is possible to increase expression by altering codon usage to prevent secondary structure at the 5' of genes [11], several alternate methods exist that can accomplish this same goal *in vivo*, such as optimizing the ribosome binding site to increase translation initiation and maximize ribosome coverage of the mRNA (and, by extension, reducing mRNA secondary structure), or by strengthening the promoter to increase mRNA levels to offset diminished protein production per mRNA. Furthermore, destabilizing 5' mRNA structure need not require significant synonymous substitutions to rare codons. The 5' synonymous nucleotide sequences generated by random reverse translations formed on average more stable secondary structures than the wild type sequences, suggesting that selective pressure against mRNA secondary structures might exist. Yet wild type mRNA sequences have on average less secondary structure stability than randomly generated sequences, without a significant change in the distribution between %Min and %Max. Hence it appears that the selective pressure against 5' secondary structure can be resolved without invoking a significant increase in rare codons. It appears that the introduction of a few synonymous codons, rare or not, is sufficient to destabilize mRNA 5' secondary structure. As a result, we conclude that the presence of significant clusters of rare codon clusters at 5' gene termini is not linked to the elimination of secondary structure, but instead to other possible functional effects.

In contrast to the 5' end, few published hypotheses exist to explain the increase in rare codon clusters observed at the 3' terminus. Some proteins, such as tailspike from *S. typhimurium *phage P22, have been shown to dwell on the ribosome post-translationally [13]. If dwelling on the ribosome aids in tailspike folding, it is possible that a 3' cluster of rare codons, which would give proteins that fold slowly additional time to fold before release from the ribosome, could replicate this mechanism. Codon usage can be altered without any constraints on the underlying amino acid sequence, which would allow any potential sequence to prolong its association with the ribosome without relying on a potentially sequence-dependent interaction with the ribosomal surface. Pausing at the C-terminus of the nascent polypeptide could also allow co-factors or chaperones to bind to the nearly complete sequence of the nascent polypeptide. It has also been suggested that 3' rare codons could serve as a signal for tagging by SsrA, the absence of which has been shown to negatively impact the expression of certain genes [36].

In conclusion, rare codon clusters are non-randomly localized and enriched at *E. coli *gene termini. Moreover, similar terminal enrichment was detected for numerous other prokaryotic organisms, and across diverse protein types, indicating potential functional roles for rare codons in protein biogenesis, folding, secretion and interactions with partner proteins.

## Methods

### %MinMax calculation

The %MinMax calculation was performed as described previously [6] using a window size of 18 and codon usage frequencies derived from the ORFeome files (described below). An open-access online %MinMax interface is available [15]. Briefly, for the *j*th codon of the *i*th amino acid with *n *synonymous codons, the %MinMax algorithm calculates the difference between the actual codon usage frequency (X_*ij*_) and the average codon usage frequency (X_avg, *i*_), divided by the difference between the maximum (X_max, *i*_) or minimum (X_min, *i*_) codon usage frequency and the average codon usage value.

### Computational methods

The *E. coli *K12-MG1655 ORFeome, containing 4288 ORFs, was obtained from the JCVI CMR database [20]. The remaining prokaryotic ORFeomes (*Agrobacterium tumefaciens, Bacillus anthracis, Bacillus cereus, Bacillus subtilis*^‡^, *Bacteriodes fragilis, Bordetella pertussis, Brucella melitensis 16 M, Burkholderia sp. 383, Coxiella burnetii, Cryptococcus neoformans, Deinococcus radiodurans, Erwinia carotovora, Heliobacter pylori*^‡^, *Neisseria meningitidis, Nostoc sp PCC 7120, Pseudomonas fluorescens, Ralstonia metallidurans CH34, Salmonella entericia, Salmonella typhimurium, Shigella flexneri, Sinorhizobium meliloti, Staphylococcus aureus*^‡^, *Thermus thermophilus, Xylella fastidiosa *and *Yersinia pestis*) were also obtained from the JCVI CMR. Eukaryotic ORFeomes for *T. brucei*, *A. thaliana, C. neoformans *were obtained from the annotated databases at JCVI. The human ORFeome was taken from DFCI-CCSB at Harvard [37]. All windows that contained a non-ATGC base were eliminated. ORFs longer than 250 windows were extracted and analyzed for the presence or absence of rare codon clusters. For each position *x*, the *x*th window of ORFs was considered to be a rare codon cluster if the %Min value was at least -10%Min, the point where enrichment of rare codons becomes statistically significant in *E. coli *[6]. The threshold was increased for organisms where -10%Min was not statistically significant to a %Min value that was statistically significant. The three ORFeomes that did not have any statistically significant %Min values (^‡^) were evaluated using the 10%Min threshold. Codon-biased random reverse translations were created by generating synonymous gene sequences composed of synonymous codons randomly selected using a table weighted for codon usage frequency.

### 5' mRNA structure calculations

The minimum folding energies (ΔG_folding_) for the first 40 nucleotides from each of the 4288 *E. coli *ORFs were calculated using UNAFold [38] with the default setting of 37°C.

### Regression analysis

For each mRNA ΔG_folding _value, the population count of ORFs with rare codon clusters was paired with the population count of ORFs without rare codon clusters. These paired values were graphed and a linear regression was performed, leading to a correlation coefficient of 0.73. To account for the small difference in the median stability of sequences with versus without rare codon clusters, the regression was repeated with offsets between the data sets ranging from +2 kcal/mol to -2 kcal/mol in increments of 0.1 kcal/mol. An offset of -0.4 kcal/mol, consistent with the differences in the medians, produced the maximum correlation coefficient (0.7945) and is the value reported in the text.

### Statistics of rare codon cluster enrichment

To determine whether rare codons were significantly enriched at gene termini for certain types of proteins, populations with or without a certain characteristic, i.e., a predicted signal sequence, were evaluated for the presence or absence of either 5' or 3' rare codon clusters. A p-value was calculated from a 2 × 2 contingency table using a Fischer's exact, two-tailed test.

### Expression level determinations

The expression level data for wild-type K12 *E. coli *were obtained from the NCBI Gene Expression Omnibus, accession numbers GSE1730 and GSE1735. The Cy5/Cy3 ratio, representing the mRNA abundance divided by the genome DNA reference, was used to rank expression levels. The eight separate datasets using wild-type cells grown in LB media were averaged together. The 530 genes with an average Cy5/Cy3 ratio greater than 2.0 were used for the highly expressed dataset while the 527 genes with an average Cy5/Cy3 ratio less than 1.15 were used for the least expressed dataset.

## Abbreviations

ΔG_folding_: the measured ΔG of folding for the RNA secondary structure as determined by UNAFold; JCVI CMR: J. Craig Venter Institute Comprehensive Microbial Resource, formerly TIGR (The Institute of Genomic Research) CMR; ORF: open reading frame; ORFeome: a collection of all the ORFs for a particular organism.

## Authors' contributions

PLC conceived of the study, and participated in its design and execution. TFC performed all computational and statistical analyses. TFC and PLC wrote the manuscript. The final version of the manuscript was read and approved by both authors.

## Supplementary Material

Additional file 1 **The abundance of specific codons in genes with terminal rare codon clusters, with signal sequences, or predicted to be secreted**. Examination of the relative codon usage for each codon at the termini of genes containing signal sequences, predicted to be secreted or with terminal rare codon clusters. Click here for file

## References

[B1] Robinson M Lilley R Little S Emtage JS Yarranton G Stephens P Millican A Eaton M Humphreys G Codon usage can affect efficiency of translation of genes in *Escherichia coli* Nucleic Acids Res 1984 12 6663 6671 10.1093/nar/12.17.6663 6091031 PMC320107

[B2] Sorensen MA Kurland CG Pedersen S Codon usage determines translation rate in *Escherichia coli* J Mol Biol 1989 207 365 377 10.1016/0022-2836(89)90260-X 2474074

[B3] Sorensen MA Vogel U Jensen KF Pedersen S The rates of macromolecular chain elongation modulate the initiation frequencies for transcription and translation in *Escherichia coli* Antonie Van Leeuwenhoek 1993 63 323 331 10.1007/BF00871227 7506514

[B4] Kane JF Effects of rare codon clusters on high-level expression of heterologous proteins in *Escherichia coli* Curr Opin Biotechnol 1995 6 494 500 10.1016/0958-1669(95)80082-4 7579660

[B5] Laughrea M Speed-accuracy relationships during in vitro and in vivo protein biosynthesis Biochimie 1981 63 145 168 10.1016/S0300-9084(81)80189-7 7013826

[B6] Clarke TF IV Clark PL Rare codons cluster PLoS One 2008 3 e3412 10.1371/journal.pone.0003412 18923675 PMC2565806

[B7] Gu W Zhou T Ma J Sun X Lu Z Folding type specific secondary structure propensities of synonymous codons IEEE Trans Nanobioscience 2003 2 150 157 10.1109/TNB.2003.817024 15376949

[B8] Makhoul CH Trifonov EN Distribution of rare triplets along mRNA and their relation to protein folding J Biomol Struct Dyn 2002 20 413 420 12437379 10.1080/07391102.2002.10506859

[B9] Thanaraj TA Argos P Protein secondary structural types are differentially coded on messenger RNA Protein Sci 1996 5 1973 1983 10.1002/pro.5560051003 8897597 PMC2143259

[B10] Thanaraj TA Argos P Ribosome-mediated translational pause and protein domain organization Protein Sci 1996 5 1594 1612 10.1002/pro.5560050814 8844849 PMC2143486

[B11] Kudla G Murray AW Tollervey D Plotkin JB Coding-sequence determinants of gene expression in *Escherichia coli* Science 2009 324 255 258 10.1126/science.1170160 19359587 PMC3902468

[B12] Zalucki YM Beacham IR Jennings MP Biased codon usage in signal peptides: a role in protein export Trends Microbiol 2009 17 146 150 10.1016/j.tim.2009.01.005 19307122

[B13] Clark PL King J A newly synthesized, ribosome-bound polypeptide chain adopts conformations dissimilar from early in vitro refolding intermediates J Biol Chem 2001 276 25411 25420 10.1074/jbc.M008490200 11319217

[B14] Hayes CS Bose B Sauer RT Stop codons preceded by rare arginine codons are efficient determinants of SsrA tagging in *Escherichia coli* Proc Natl Acad Sci USA 2002 99 3440 3445 10.1073/pnas.052707199 11891313 PMC122542

[B15] %MinMax Calculator http://www.codons.org

[B16] Sharp PM Li WH The Codon Adaptation Index--a measure of directional synonymous codon usage bias, and its potential applications Nucleic Acids Res 1987 15 1281 1295 10.1093/nar/15.3.1281 3547335 PMC340524

[B17] Zhang G Ignatova Z Generic algorithm to predict the speed of translational elongation: implications for protein biogenesis PLoS One 2009 4 e5036 10.1371/journal.pone.0005036 19343177 PMC2661179

[B18] Parmley JL Huynen MA Clustering of codons with rare cognate tRNAs in human genes suggests an extra level of expression regulation PLoS Genet 2009 5 e1000548 10.1371/journal.pgen.1000548 19578405 PMC2697378

[B19] Sorensen MA Pedersen S Absolute in vivo translation rates of individual codons in *Escherichia coli*. The two glutamic acid codons GAA and GAG are translated with a threefold difference in rate J Mol Biol 1991 222 265 280 10.1016/0022-2836(91)90211-N 1960727

[B20] Peterson JD Umayam LA Dickinson T Hickey EK White O The Comprehensive Microbial Resource Nucleic Acids Res 2001 29 123 125 10.1093/nar/29.1.123 11125067 PMC29848

[B21] Kowarik M Kung S Martoglio B Helenius A Protein folding during cotranslational translocation in the endoplasmic reticulum Mol Cell 2002 10 769 778 10.1016/S1097-2765(02)00685-8 12419221

[B22] Malkin LI Rich A Partial resistance of nascent polypeptide chains to proteolytic digestion due to ribosomal shielding J Mol Biol 1967 26 329 346 10.1016/0022-2836(67)90301-4 4962271

[B23] Purvis IJ Bettany AJ Santiago TC Coggins JR Duncan K Eason R Brown AJ The efficiency of folding of some proteins is increased by controlled rates of translation in vivo. A hypothesis J Mol Biol 1987 193 413 417 10.1016/0022-2836(87)90230-0 3298659

[B24] Namy O Moran SJ Stuart DI Gilbert RJ Brierley I A mechanical explanation of RNA pseudoknot function in programmed ribosomal frameshifting Nature 2006 441 244 247 10.1038/nature04735 16688178 PMC7094908

[B25] Katz L Burge CB Widespread selection for local RNA secondary structure in coding regions of bacterial genes Genome Res 2003 13 9 2042 2051 10.1101/gr.1257503 12952875 PMC403678

[B26] Bendtsen JD Nielsen H von Heijne G Brunak S Improved prediction of signal peptides: SignalP 3.0 J Mol Biol 2004 340 783 795 10.1016/j.jmb.2004.05.028 15223320

[B27] Bendtsen JD Jensen LJ Blom N Von Heijne G Brunak S Feature-based prediction of non-classical and leaderless protein secretion Protein Eng Des Sel 2004 17 349 356 10.1093/protein/gzh037 15115854

[B28] Edgar R Domrachev M Lash AE Gene Expression Omnibus: NCBI gene expression and hybridization array data repository Nucleic Acids Res 2002 30 207 210 10.1093/nar/30.1.207 11752295 PMC99122

[B29] Rodnina MV Wintermeyer W Recent mechanistic insights into eukaryotic ribosomes Curr Opin Cell Biol 2009 21 435 443 10.1016/j.ceb.2009.01.023 19243929

[B30] Cortazzo P Cervenansky C Marin M Reiss C Ehrlich R Deana A Silent mutations affect in vivo protein folding in *Escherichia coli* Biochem Biophys Res Commun 2002 293 537 541 10.1016/S0006-291X(02)00226-7 12054634

[B31] Kumada Y Sakan Y Kajihara H Kihara M Kikuchi Y Yamaji H Seong GH Katoh S Efficient production of single-chain Fv antibody possessing rare codon linkers in fed-batch fermentation J Biosci Bioeng 2009 107 73 77 10.1016/j.jbiosc.2008.09.001 19147114

[B32] Kimchi-Sarfaty C Oh JM Kim IW Sauna ZE Calcagno AM Ambudkar SV Gottesman MM A "silent" polymorphism in the MDR1 gene changes substrate specificity Science 2007 315 525 528 10.1126/science.1135308 17185560

[B33] Widmann M Clairo M Dippon J Pleiss J Analysis of the distribution of functionally relevant rare codons BMC Genomics 2008 9 207 10.1186/1471-2164-9-207 18457591 PMC2391168

[B34] Lakkaraju AK Mary C Scherrer A Johnson AE Strub K SRP keeps polypeptides translocation-competent by slowing translation to match limiting ER-targeting sites Cell 2008 133 440 451 10.1016/j.cell.2008.02.049 18455985 PMC2430734

[B35] Brandt F Etchells SA Ortiz JO Elcock AH Hartl FU Baumeister W The native 3D organization of bacterial polysomes Cell 2009 136 261 271 10.1016/j.cell.2008.11.016 19167328

[B36] Abo T Inada T Ogawa K Aiba H SsrA-mediated tagging and proteolysis of LacI and its role in the regulation of lac operon EMBO J 2000 19 3762 3769 10.1093/emboj/19.14.3762 10899129 PMC313975

[B37] Lamesch P Li N Milstein S Fan C Hao T Szabo G Hu Z Venkatesan K Bethel G Martin P hORFeome v3.1: a resource of human open reading frames representing over 10,000 human genes Genomics 2007 89 307 315 10.1016/j.ygeno.2006.11.012 17207965 PMC4647941

[B38] Markham NR Zuker M DINAMelt web server for nucleic acid melting prediction Nucleic Acids Res 2005 33 W577 581 10.1093/nar/gki591 15980540 PMC1160267

