# Supplementary material for: Increased incidence of rare codon clusters at 5' and 3' gene termini:implications for function
Source: BMC Genomics. 2010 Feb 18;11:118. doi: 10.1186/1471-2164-11-118 (PMC2833160; doi:10.1186/1471-2164-11-118)
Supplement: Additional file 1 — The abundance of specific codons in genes with terminal rare codon clusters, with signal sequences, or predicted to be secreted. Examination of the relative codon usage for each codon at the termini of genes containing signal sequences, predicted to be secreted or with terminal rare codon clusters. [file 1471-2164-11-118-S1.DOC]

Increased incidence of rare codon clusters at 5’ and 3’ gene termini:

Implications for function

Thomas F. Clarke IV and Patricia L. Clark*

Department of Chemistry and Biochemistry

University of Notre Dame, Notre Dame IN, 46556

**Additional Information**


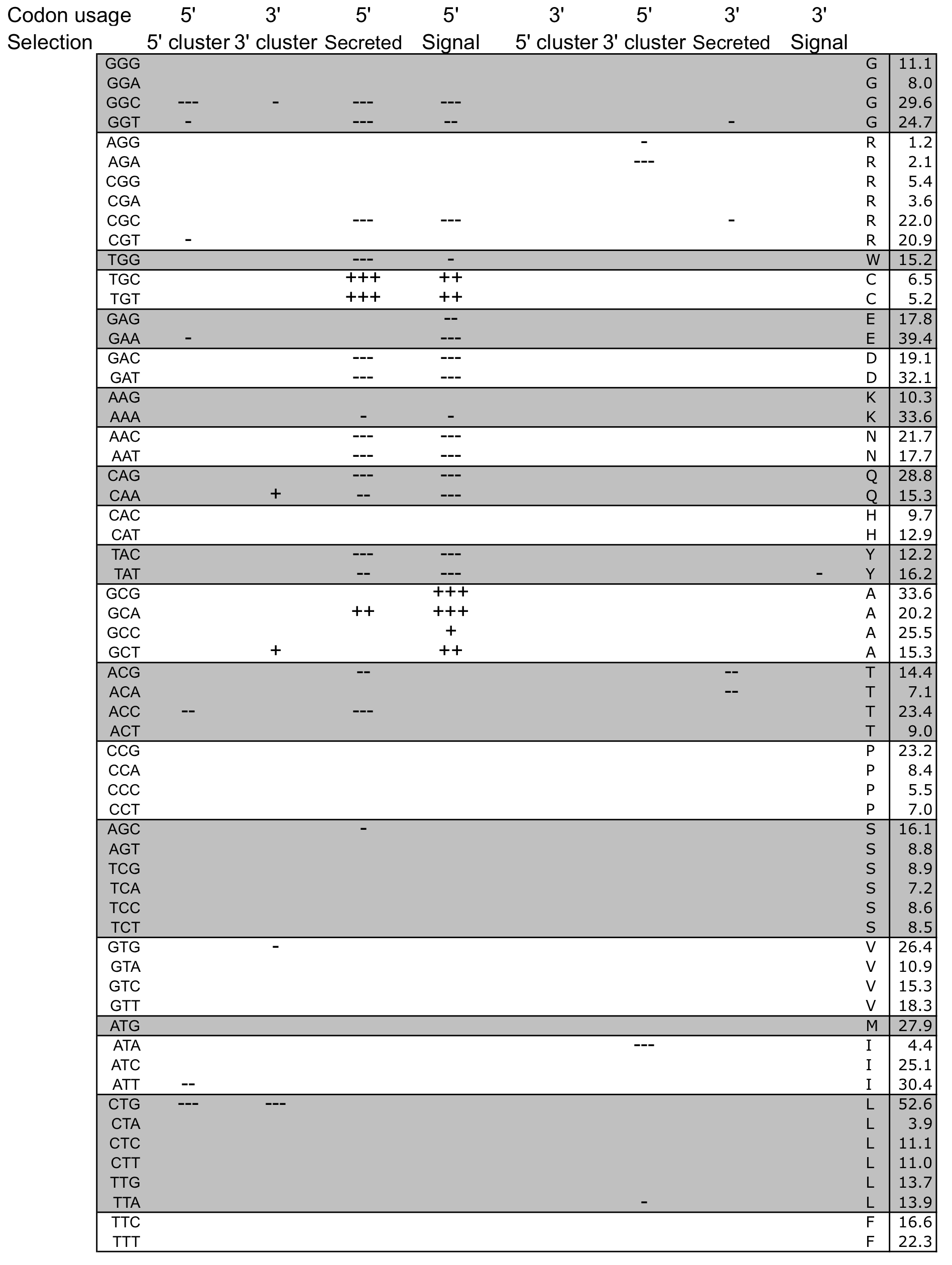


**Figure S1.** The abundance of specific codons in genes with terminal rare codon clusters, with signal sequences, or predicted to be secreted. The codon usage for each termini of genes was determined and statistically significant enrichment (+) or under-enrichment (-) of each codon is shown (+/- for p < 1e-04, ++/-- for p < 1e-06, +++/--- for p < 1e-08). As a control, the codon usage at the opposite terminus, for example, the 3’ codon usage for genes with 5’ rare codon clusters, was also calculated. The total codon usage per 1000 codons for *E. coli* is shown in the right-hand column. The 5' codon usage for genes predicted to be secreted or predicted to have a signal sequence shows statistically significant variation; however, this is primarily related to amino acid usage, not selection between codons. Tryptophans, aspartic acid, asparagine, glutamine and tyrosine are all under-represented in signal sequences, while cysteines and alanines are over-represented. There is under-enrichment of the most common glycine, arginine and threonine codons, though no rare codons are specifically over-enriched.

**Supplementary Methods**

*Determination of enrichment or underenrichment of specific codons.* For 5' rare codon clusters, genes were separated into two groups: those with 5' rare codon clusters and those without 5' rare codon clusters. The codon usage for the first 50 codons, the final 50 codons and the interior codons was tallied for each group. Two separate 2x2 contingency tables were then constructed, using the terminal codon usage (5' in one table, 3' in the other) and interior codon usage as columns and the genes with 5' rare codon clusters and without 5' rare codon clusters as rows. A chi-square with Yates correction was used to calculate the p-value for the distribution. This process was then repeated for genes with 3' rare codon clusters, predicted signal sequences and predicted secreted genes.
